# Fos expression in the brains of rats performing an attentional set-shifting task

**DOI:** 10.1016/j.neuroscience.2010.09.008

**Published:** 2010-12-01

**Authors:** K.E. Burnham, D.M. Bannerman, L.A. Dawson, E. Southam, T. Sharp, M.G. Baxter

**Affiliations:** a Department of Pharmacology, University of Oxford, Mansfield Road, Oxford, UK; b Department of Experimental Psychology, University of Oxford, South Parks Road, Oxford, UK; c Psychiatry Centre for Excellence in Drug Development, Glaxo Smith Kline, New Frontiers Science Park, Third Avenue, Harlow, Essex, UK

**Keywords:** Fos, prefrontal cortex, intradimensional shift, extradimensional shift, executive function, CD, compound discrimination, ED, extradimensional, Fos-LI, Fos-like immunoreactivity, GAD_67_, Glutamic acid decarboxylase 67, ID, intradimensional, IDY, ID yoked, IE, intermediate entorhinal, IEG, immediate-early gene, LE, lateral entorhinal, ME, medial entorhinal, mPFC, medial prefrontal cortex, OFC, orbital frontal cortex, PBS, phosphate buffered saline, PFC, prefrontal cortex, PPC, posterior parietal cortex, PRh, perirhinal cortex, Rev, reversal, SD, simple discrimination

## Abstract

Impairments in executive function and cognitive control are a common feature of neuropsychiatric and neurodegenerative disorders. A promising behavioral paradigm for elucidating the neural mechanisms of executive function is extradimensional/intradimensional (ED/ID) shifting, which places demands on executive function by requiring the adjustment of behavioral responses based on affective or attentional information. To augment the understanding of the brain systems required for these aspects of executive function, we examined the induction of Fos protein in rats tested in the ED/ID paradigm. We found increased Fos-like immunoreactivity (Fos-LI) in several cortical areas, including medial and orbital frontal cortex (OFC), in rats performing affective or attentional shifts relative to rats performing control discriminations. However, increased Fos-LI was also present in rats that performed a yoked number of additional control discrimination trials, without affective or attentional shifting. These observations suggest that cortical networks required for affective and attentional shifting are also activated during comparable discrimination tasks that do not require shifting, consistent with a role for these networks in monitoring ongoing behavior even in situations in which adaptation to changing behavioral demands is not required.

The ability to direct attention towards relevant and away from irrelevant stimuli in the environment is a core component of executive control mechanisms in cognition. Another is the capacity to flexibly alter associations between stimuli, actions, and reinforcers, so that when reinforcement contingencies change, behavior changes in parallel. These two processes, referred to as “attentional” and “affective” shifting, respectively, are dependent on different frontal cortex subregions (Dias et al., 1996; Birrell and Brown, 2000; McAlonan and Brown, 2003; Bissonette et al., 2008), plus other cortical regions (Oswald et al., 2001; Fox et al., 2003) and specific neuromodulatory systems, particularly noradrenaline (Lapiz and Morilak, 2006; Tait et al., 2007; McGaughy et al., 2008). Impairments in attentional and affective shifting, as well as in other aspects of executive function and cognitive control, are common to many neurodegenerative and neuropsychiatric disorders, including Parkinson's disease, schizophrenia, and obsessive-compulsive disorder (Owen et al., 1992; Veale et al., 1996; Weickert et al., 2000).

Affective and attentional shifting can be assessed in a behavioral protocol involving discrimination between pairs of discriminanda composed of two or more sensory dimensions (e.g. color and shape) presented in sequence. Different discriminations are presented, using novel stimuli for each, in which the same sensory dimension is relevant for solving the discrimination, establishing an “attentional set” to one of the sensory dimensions through this series of intradimensional (ID) shifts. Once learned, the reward contingencies may be reversed, to assess affective shifting as the efficiency of learning the discrimination reversal. Attentional shifting is tested by presenting new discriminations in which the relevant dimension changes from previous discriminations, referred to as an extradimensional (ED) shift. In ED shifts, the subject must attend to elements of the previously-irrelevant dimension to solve the discrimination while ignoring the components of the previously-relevant dimension. Critically, in these discriminations all stimuli are novel, so that slower acquisition of ED shifts relative to ID shifts reflects a demand on attentional processes rather than a tendency to respond to previously-rewarded stimuli.

Event-related functional imaging studies have demonstrated activation of the lateral prefrontal cortex (PFC) in human and non-human primates during ED set-shifting and reversal learning relative to ID shifting (Konishi et al., 1999; Rogers et al., 2000; Nakahara et al., 2002; Hampshire and Owen, 2006). In addition, there is evidence of distinct neural circuit activation, involving a cortico-basal ganglia loop and an occipito-temporal pathway, during ED set-shifting (Rogers et al., 2000; Monchi et al., 2001). However, limited data are available about the pattern of cerebral activations during attentional and affective shifting in rodents. Such data would complement existing lesion data in the ED/ID procedure, and may provide insights into whether specific cell populations within cortical areas are engaged by attentional or affective shifting. To investigate the neuroanatomical basis of attentional and affective shifting in rodents, we measured expression of an immediate-early gene (IEG), Fos, that is associated with neuronal activation and plasticity (Sagar et al., 1988; Dragunow et al., 1989) after training rats on discrimination problems concluding with attentional or affective shifts. We expected to find differential Fos-like immunoreactivity (Fos-LI) in cortical areas identified on the basis of lesion studies, for example medial and orbital PFC, posterior parietal cortex (PPC) and entorhinal cortex, in rats that had performed an attentional shift compared to rats that had not.

We performed three experiments to test this hypothesis. All of these involved training groups of rats on a sequence of discrimination problems that was identical between the various groups, with the critical manipulation presented in the final discrimination, given 90 min after the beginning sequence. This was to allow induction of Fos in response to discrimination training to return to baseline levels, so that Fos protein determined at sacrifice would primarily reflect neuronal activity in response to the final phase of discrimination learning. First, we compared Fos expression in the brains of rats whose final discrimination was an ED shift to that in brains of rats whose final discrimination was an ID shift (Experiment 1). Both groups of rats therefore encountered novel stimuli in their final discrimination, but one group had to shift attention to a new stimulus dimension to solve the problem efficiently and the other did not. As we point out in our discussion of these results, there is a limitation in this design in that rats find the ID shift easier than the ED shift, so there is a difference in the number of trials worked, rewards earned, and so forth between rats in these groups. Nevertheless, this is similar in design to other studies that have examined gene expression in response to learning in these kinds of tasks, where comparisons are made between gene expression at the end of an earlier stage of training (the compound discrimination (CD)) to the ED shift (Glickstein et al., 2005; DeSteno and Schmauss, 2008). Thus, although there is a potential weakness in the experimental design, this experiment does allow us to compare Fos expression in response to our behavioral training, using our methods for Fos determination and quantification, to that observed in other studies.

A similar design was used to examine Fos expression in response to reversal learning (Experiment 2). The design was identical to Experiment 1, except the final phase was a reversal of the most recently-learned discrimination, or a repeat of that discrimination problem without reversal learning. In this experiment, because we were mindful that a repeat of the discrimination would involve no new learning, we chose to yoke the number of repeated trials of individual rats in this group to those of rats in the group undergoing reversal learning. Experiment 3 was a replication of Experiment 1, but we included an additional group of rats that underwent an ID shift in the final phase of training and completed a yoked number of trials to rats in the ED shift group. This allowed us to address the potential flaw in the design of Experiment 1 (shared by all foregoing studies using this paradigm to investigate behavior-induced gene expression).

As a secondary goal of this research, we used double-labelling immunohistochemistry with Glutamic acid decarboxylase 67 (GAD_67_) to determine whether neurons activated by attentional set-shifting were GABA-ergic in nature. Dysfunction of GABAergic neurons in the PFC, specifically reductions in the calcium binding protein parvalbumin and the GABA synthesising enzyme GAD_67_, have been identified in patients with schizophrenia (Reynolds et al., 2001; Volk and Lewis, 2002), and the resulting GABAergic dysfunction may be an important contributor to PFC dysfunction and associated cognitive deficits (Lewis et al., 2005). In rats, chronic intermittent administration of phencyclidine reduced parvalbumin mRNA in the medial prefrontal cortex (mPFC; Pratt et al., 2008) and produced deficits in attentional set-shifting (Rodefer et al., 2005; Pratt et al., 2008). These data suggest that a functional GABA-ergic system may be important for attentional set-shifting.

## Experimental procedures

### Subjects

Adult male Lister hooded rats weighing 200–300 g at the start of testing were group housed in a temperature and humidity controlled environment (21±1 °C), lights on 0700–1900 h, with water freely available. Before behavioral testing, all rats, including rats in control groups, were placed on a restricted feeding schedule and maintained at 85–90% of their free-feeding weight throughout experiments. All studies were performed in accordance with the terms of the UK Animals (Scientific Procedures) Act 1986, and its associated guidelines, under the authority of personal and project licences from the UK Home Office. All efforts were made to minimize the suffering and number of animals used.

### Behavioral testing

#### Apparatus

The test chamber consisted of a modified plastic home cage (40×70×18 cm^3^) with Perspex dividers separating the cage into a large start chamber (40×46 cm^2^) and two identically sized (24×20 cm^2^) choice chambers to which access was controlled by removable Perspex doors. The digging bowls were ceramic (internal diameter 7 cm, depth 4 cm, Mason and Cash, UK) and were placed within the choice chambers. The bowls were filled with digging media of different textures and the media were scented with different herbs and spices (see Table 1 for examples). The bowls were baited with Honey Nut Cheerios (Nestle, Surrey, UK), with ground up Honey Nut Cheerios mixed with the digging medium to serve as an odour mask. During testing only one of the bowls was baited and rats were required to determine which of the two bowls was baited using either the texture of the digging medium or the odour of the medium as cues.

#### Habituation

Rats were habituated to the apparatus over 5 days, and taught to dig in the ceramic bowls filled with home-cage bedding to receive one quarter of a Honey Nut Cheerio. On the day before testing, rats performed two simple discriminations, one based on odour (lavender vs. lemon bedding) and one based on medium (small clay particles vs. large clay particles), to a criterion of six consecutive correct trials. This was to ensure that the rats could learn to discriminate between the two bowls to receive a food reward. The order of the training discriminations (odour and medium) and the rewarded odour and digging medium were counterbalanced, and the exemplars were not used again throughout testing. Digging was defined as active digging with both front paws or active foraging with the snout in the digging medium. Sniffing or touching the medium with the paws was not scored as a dig.

#### Testing paradigm

The ED/ID paradigm (Birrell and Brown, 2000) had previously been used within this laboratory by Tunbridge et al. (2004). However, to examine the effects specific elements of the paradigm on Fos protein expression, it was necessary to make a number of modifications. Firstly, the discriminations were arranged so that the discrimination of interest (e.g. ED, ID or reversal (Rev) discrimination) was at the end of the discrimination series. Secondly, a 90 min break was introduced between the first four discriminations and the final discrimination. During this break rats were isolated in a dark room to which they had been habituated previously. This was to ensure that, as far as possible, any changes in Fos expression observed were as a result of the final discrimination and not a result of the acquisition of the first four discriminations. Because the effect of reversal learning on Fos expression was to be examined, reversal discriminations were excluded from the initial stages of the task to prevent any possible practice effect. An additional ID shift was introduced to help reinforce the attentional set.

All the discriminations in the testing sequence were presented in a single test day. The first four trials of each discrimination were “discovery” trials, during which the rat was allowed to dig in both bowls, regardless of where he first began to dig, to discover which bowl contained the reward. An error was recorded if the rat dug first in the unbaited bowl, but these trials were not included in the trials or errors to criterion score. After the first four trials, if the rat dug in the unbaited bowl, an error was recorded and access to the correct bowl was denied. The rat returned to the start chamber of his own accord to start the next trial. Testing continued until the rat reached a criterion of six correct consecutive trials.

The sequence of discriminations included a simple discrimination (SD) between either two odours or two digging mediums, followed by a CD which had the same positive stimulus as the SD, but now also involved a new irrelevant dimension which was not a reliable predictor of the location of the reward. This was followed by two ID shifts, which were CDs in which both the relevant and irrelevant stimuli changed, but the relevant dimension (either odour or medium) remained the same. Depending on the group to which the rat had been allocated, there was then a third ID shift, an ED shift or a reversal. In the ED shift, the relevant and irrelevant stimuli changed, but also the relevant dimension changed, so if odour had previously predicted the location of the reward, now digging medium would become the relevant dimension. In a Rev discrimination all of the stimuli remained the same as in the previous discrimination, and the relevant dimension remained the same, but the rewarded and non-rewarded stimuli within the dimension were reversed. The particular sequence of discriminations, including the final discrimination, depended on the particular experiment, as follows:

Experiment 1: Two groups of rats (*n*=8 per group) performed a series of discriminations in the following order: SD, CD, ID, ID2, then either ID3 or ED depending on the group to which they had been assigned.

Experiment 2: Two groups of animals (*n*=8 per group) performed the SD, CD, ID, and ID2 discriminations, then one group performed a discrimination in which the rewarded exemplars from ID2 were reversed (i.e. Rev discrimination), while the other group repeated ID2 (IDrep, i.e. a repeat of ID2). It was important that the exemplars were not novel in the IDrep discrimination since the exemplars in the Rev discrimination were not novel. Once the animals performing the IDrep had completed six consecutive correct trials, they continued performing trials until they had completed the same number as one of the animals performing the Rev discrimination. In this way, the IDrep group were yoked to the Rev group in terms of number of trials completed, to control for the effects of the number of trials performed on Fos expression.

Experiment 3: The third experiment was a replication of Experiment 1, except that a third group of animals were included which performed the ID3, but were yoked to the ED group in terms of number of trials completed. This group is referred to as ID yoked (IDY).

An example of the odour and medium exemplars used as the dimensions to be discriminated are shown in Table 1. Each pair of digging media were made from the same material to ensure that the rats could not use odour to differentiate between digging media when texture was the relevant dimension. Assignment of animals to groups was random, but groups were counterbalanced for the rewarded stimulus within a pair, shift direction at the ED stage (odour to medium or medium to odour) and the sequence in which the animals encountered the pairs of stimuli.

After completion of the ID3/IDY/ED/Rev the rats were placed in the dark environment for 120 min before being killed by barbiturate overdose and transcardial perfusion. In addition to the animals that underwent behavioral testing, additional groups of cage-mate controls were included in Experiments 2 and 3. These rats had the same exposure to the dark environment as the rats in the other conditions, but were behaviorally naive and remained in their home-cage before being transferred to the dark environment.

### Fos immunohistochemistry

Two hours after completion of behavioral testing, animals were deeply anaesthetized with pentobarbitone (300 mg/kg i.p.; Animalcare Ltd., York, UK) and transcardially perfused with 0.9% saline followed by 250 ml 4% paraformaldehyde in phosphate buffered saline (PBS: 140 mM NaCl, 30 mM KCl, 80 mM Na_2_HPO_4_, 15 mM KH_2_PO_4_, in distilled water) with 0.4% picric acid. Brains were removed and postfixed overnight (4 °C), then incubated in 30% sucrose over 48–72 h (4 °C). Free-floating coronal sections (40 μm) were cut on a freezing microtome, the slices were then preserved in cryoprotectant (PBS containing 24% glycerol (v/v) and 24% ethylene glycol (v/v) at −20 °C) and stored at −20 °C until processing for immunohistochemistry.

For Fos immunohistochemistry, all sections were counter-stained for GAD_67_ immunoreactivity to facilitate cell identification. Sections were washed in PBS, incubated in hydrogen peroxide (0.3%, 10 min), washed in PBS again, treated (30 min) with standard blocking serum (10% normal goat serum in PBS) then incubated overnight (4 °C) in mouse anti-GAD_67_ primary antibody (#MAB5406, Chemicon International, Hampshire, UK, 1:2000 dilution) in 10% NGS-PBS with gentle agitation. Sections were then washed in PBS and incubated (2 h) in goat anti-mouse biotinylated secondary antibody (#BA9200, Vector Laboratories Ltd., Peterborough, UK, 1:500 dilution). GAD_67_ immunoreactivity was visualized using a chromagen reaction to give a brown product (ABC Elite Kit and DAB kit, Vector Laboratories Ltd., Peterborough, UK). Sections were washed in PBS before incubation (72 h at 4 °C with gentle agitation) with rabbit anti-c-Fos primary antibody (#sc-253, Santa Cruz Biotechnology Inc., Wiltshire, UK, 1:2000 dilution), followed by goat anti-rabbit biotinylated secondary antibody (#BA1000, Vector Laboratories Ltd., Peterborough, UK, 1:500 dilution). Fos-like immunoreactivity (Fos-LI) was visualized using a chromagen reaction to give a blue-black product (ABC Elite Kit and SG kit, Vector Laboratories Ltd., Peterborough, UK).

### Immunohistochemical data collection

**Fig. 1:**
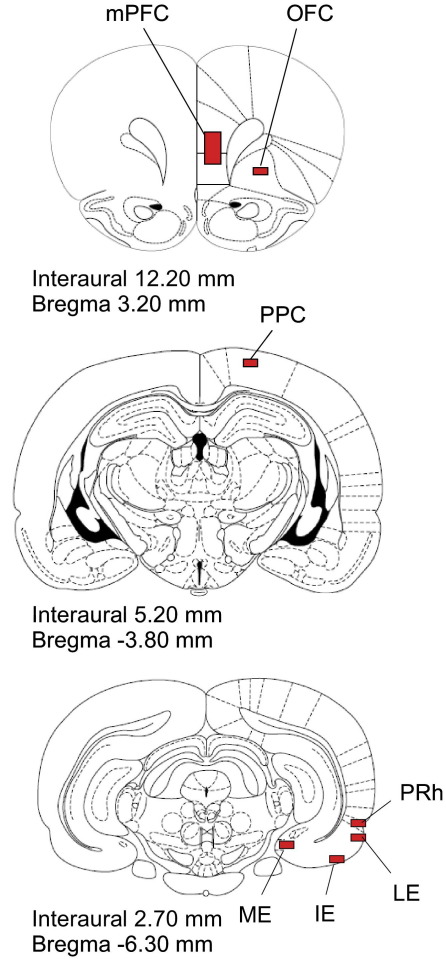
Diagrammatical representation of regions in which number of Fos-positive cells were counted. mPFC, medial prefrontal cortex; OFC, orbital prefrontal cortex; PPC, posterior parietal cortex; ME, medial entorhinal cortex; IE, intermediate entorhinal cortex; LE, lateral entorhinal cortex; PRh, perirhinal cortex. As defined by Paxinos and Watson (1997). For interpretation of the references to color in this figure legend, the reader is referred to the Web version of this article.

Counts of Fos-positive nuclei and Fos/GAD_67_ double-labelled cells were taken from areas previously found to be involved in attentional or affective shifting. These included areas of the mPFC; mean counts from the prelimbic and infralimbic areas, Birrell and Brown, 2000), orbital frontal cortex (OFC, McAlonan and Brown, 2003), (PPC, Fox et al., 2003), entorhinal cortex (Oswald et al., 2001), and perirhinal cortex (PRh) as defined by Paxinos and Watson (1997). The entorhinal cortex was further subdivided into the medial (ME), intermediate (IE) and lateral (LE) areas as defined by Insausti et al. (1997) (Fig. 1). Fos immunoreactive cells were counted manually by an observer blind to treatment, using a light microscope (Leitz Diaplan; 390×280 μm^2^ grid in eyepiece of ×25 objective). Double-counting of the same cell was minimized through the use of thin sections (40 μm) and non-consecutive section collection (every fourth section). Three bilateral sections per brain region per animal were counted and averaged to give number of Fos positive cells or the number of Fos/GAD_67_ double-labelled cells per mm^2^ of tissue. These methods were chosen based on those used by Aggleton and colleagues for IEG quantification (Shires and Aggleton, 2008; Poirier and Aggleton, 2009; Albasser et al., 2010). Because the number of Fos/GAD_67_ double-labelled cells in Experiment 1 was very low these data were not analysed in subsequent experiments. Because we were interested in comparing relative levels of Fos expression within particular brain areas between different behavioral conditions, which we did not expect to alter size or number of neuronal nuclei, we did not employ stereological counting methods (see Saper, 1996 for further discussion of these methods).

### Statistical analysis

In the behavioral experiments, all animals completed the first four discriminations in an identical manner, therefore the number of trials taken to reach the criterion of six correct consecutive trials on these discriminations were analysed using a one way ANOVA with repeated measures to ensure that all groups were performing comparably. In experiments 1 and 2, the number of trials and errors taken to reach criterion on the ID3 and ED discriminations and the IDrep and Rev discriminations were compared between subjects using unpaired *t*-tests. In Experiment 2 the number of trials and errors taken to reach criterion on the ID2 and the Rev discriminations were compared within subjects using a paired *t*-test. In Experiment 3, the number of trials taken to reach criterion on the ID3, IDY and ED discriminations were compared between subjects using a one way ANOVA followed by Duncan's post hoc tests.

The total number of Fos positive cells per mm^2^ and the number of Fos/GAD_67_ double-labelled cells in seven brain regions were analysed according to the behavioral discrimination performed using a two-way ANOVA with repeated measures (between subjects factor: behavioral group, within subjects repeated measure: brain region). Simple main effects were analysed and Duncan's post hoc tests used where appropriate. Planned comparisons based on a priori predictions were analysed using *t*-tests (one-tailed).

## Results

In all behavioral experiments there were no significant differences between groups on the first four discriminations (*F*<1; *P*>0.2 in all cases) indicating that animals were matched across groups in terms of efficiency of performance.

### Experiment 1

**Fig. 2:**
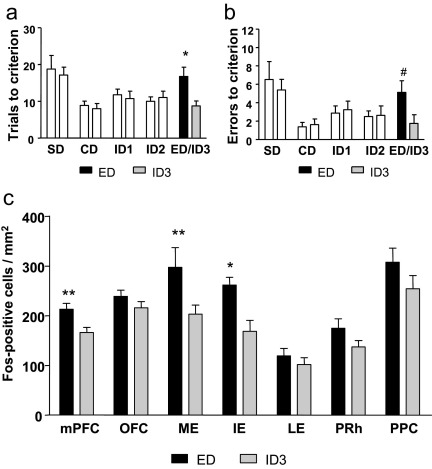
The number of trials taken (a), and number of errors made (b) to reach a criterion of six consecutive correct trials in the attentional set-shifting paradigm. Animals from each group performed identical discriminations throughout simple discrimination (SD), compound discrimination (CD) and intra-dimensional shifts 1 and 2 (ID1 and ID2). During the final discrimination animals performed either a third ID shift (ID3) or an extra-dimensional shift (ED). Data are expressed as mean±SEM, *n*=8. * *P*<0.05, ^#^*P*=0.051 different from ID3 (Student's *t*-test). (c) The mean number of Fos-positive cell counts per mm^2^ tissue in the mPFC, OFC, ME, IE, LE, PPC and PRh from animals which had performed intra-dimensional shifts (ID3) or animals which had performed EDs. Data are expressed as mean±SEM, *n*=8. * *P*<0.05, ** *P*<0.01 significantly different from ID3 (Student's *t*-test).

Rats performing an ED shift during the final stage of Experiment 1 took significantly more trials to reach criterion levels of performance than rats performing an ID shift (*t*_(14)_=2.78; *P*<0.05) (Fig. 2a). Rats also made more errors on the ED shift than the ID shift, but this narrowly missed reaching statistical significance (*t*_(14)_=2.13; *P*=0.051) (Fig. 2b). Importantly, this validates the ED shift as a test of shifting of perceptual attentional set (Birrell and Brown, 2000) and confirms that the modifications to the discrimination paradigm—specifically the omission of reversals and a 90 min break before the final discrimination—did not adversely affect the formation or retention of an attentional set.

Fos-positive cell counts were higher overall in brain regions sampled from rats that had performed the ED shift relative to the ID shift. This difference appeared to be magnified in some brain areas that are known to be critical for ED shift performance. Overall, there was a significant effect of behavioral group on Fos positive cell counts (*F*_(1,14)_=7.4 *P*<0.01), and a significant effect of brain region (*F*_(6,84)_=26.4 *P*<0.01), but no significant behavioral group × brain region interaction (*F*_(6,84)_=1.9 *P*=0.09). Although these data suggest that there was global activation of all brain regions sampled, paired comparisons were performed in different brain regions to ascertain whether statistical significance was obtained in key regions of interest, identified a priori on the basis of previous lesion studies (Birrell and Brown, 2000; Oswald et al., 2001; McAlonan and Brown, 2003). Rats which had performed an ED shift during the last phase of the task had significantly greater Fos-positive cell counts in the mPFC (*F*_(1,14)_=9.14 *P*<0.01), ME (*F*_(1,14)_=4.77 *P*<0.05) and IE (*F*_(1,14)_=11.87 *P*<0.01) compared with rats which had performed an ID shift (Fig. 2b). Differences in other brain areas were not significant in pairwise comparisons (*P*>0.13).

**Fig. 3:**
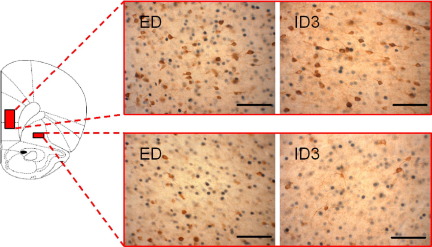
Photomicrographs of mPFC (top) and OFC (bottom) sections from rats which had completed either an ED or an intra-dimensional (ID3) discrimination. The brown stain is GAD_67_-immunolabelling, and the blue/black stain is Fos-like immunolabelling. Scale bars=100 μm.

With regard to differences in the effect of behavioral task between brain areas, we were particularly interested in the frontal cortex in which there is substantial neuropsychological evidence that the mPFC is necessary for efficient ED performance, whereas the OFC is not. A focused ANOVA comparing Fos-positive cells in these two areas indicated that the difference in Fos-positive cells between ED and ID conditions was greater for the mPFC than in the OFC. This analysis revealed effects of task and region, as expected, but critically an interaction of task and region, *F*_(1,14)_=5.62, *P*=0.033. Representative sections from the mPFC and OFC of an animal performing an ED shift and an animal performing an ID shift are presented in Fig. 3.

There was no significant effect of behavioral group on the number of Fos/GAD_67_ double-labelled cells (*F*_(1,14)_=2.0; *P*>0.1), but there was an effect of brain region (*F*_(6,84)_=36.3 *P*<0.01) and a behavioral group × brain region interaction (*F*_(6,84)_=3.74; *P*<0.01) (Table 2). Simple main effects demonstrated that the only brain region in which there was a significant effect of behavioral group was the PPC, where rats which had performed an ED shift had a greater number of Fos/GAD_67_ double-labelled cells than rats which had performed an ID shift (*F*_(1,14)_=5.26; *P*<0.05). The total number of Fos/GAD_67_ double-labelled cells was very low, averaging only 3% of the total number of Fos-positive cells across brain regions following both ED and ID discriminations, therefore these were not counted in subsequent experiments.

### Experiment 2

**Fig. 4:**
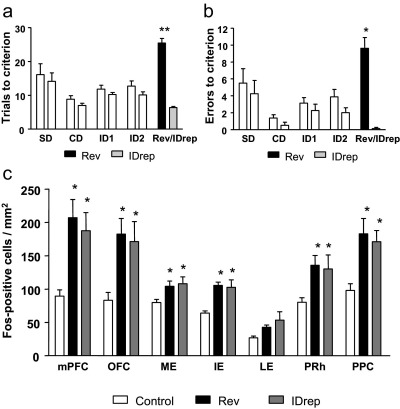
The number of trials taken (a) and number of errors made (b) to reach a criterion of six consecutive correct trials on the attentional set-shifting paradigm. Animals from each group performed identical discriminations throughout SD, CD and intra-dimensional shifts 1 and 2 (ID1 and ID2). During the final discrimination animals performed either a reversal (Rev) or a repeat of ID2 (IDrep). Note that animals which completed the IDrep then went on to complete the same number of trials as their yoked Rev animal. Data are expressed as mean±SEM, *n*=8. * *P*<0.05 and ** *P*<0.01 significantly different from IDrep (Student's *t*-test). (c) The mean number of Fos-positive cell counts per mm^2^ tissue in the mPFC, OFC, ME, IE, LE, PPC and PRh from experimentally naïve cage-mate controls, animals which had performed a reversal (Rev), or animals which had performed a repeat intra-dimensional shift (IDrep) in which the number of trials completed was yoked to the Rev animals. Data are expressed as mean±SEM, *n*=8. * *P*<0.05 significantly different from cage controls (Duncan's test), other statistics as detailed in results section.

Rats took significantly more trials and made more errors to learn the Rev discrimination than ID2 (*t*_(7)_=7.3; *P*<0.01 and *t*_(7)_=7.9; *P*<0.01 respectively). In addition, rats performing the Rev discrimination during the final stage took significantly more trials and made more errors than rats performing the IDrep discrimination, (*t*_(14)_=10.0; *P*<0.01 and *t*_(14)_=7.5; *P*<0.01, respectively) (Fig. 4a, b). Note that on reaching criterion, rats performing the IDrep discrimination then carried on to complete an equal number of trials to their yoked Rev animal.

Rats which underwent behavioral testing had significantly higher Fos-positive cell counts than behaviorally naive cage-mate controls in all brain regions other than the LE. There were no differences in Fos-positive cell counts between rats which performed the Rev discrimination and those which performed the IDrep discrimination. Thus, there was no evidence of specific activation caused by reversal learning, relative to continuing to repeat the already-learned ID discrimination without a reversal of reward contingencies. Overall, there was a significant effect of behavioral group (cage control, Rev, or IDrep, *F*_(2,21)_=11.6; *P*<0.01, and a significant effect of brain region (*F*_(6,126)_=31.0; *P*<0.01) on Fos-positive cell counts. There was also a significant behavioral group×brain region interaction (*F*_(12,126)_=2.4; *P*<0.05). Analysis of simple main effects demonstrated that there was an effect of behavioral group on Fos positive counts in the mPFC (*F*_(2,21)_=7.6; *P*<0.01), ME (*F*_(2,21)_=3.6; *P*<0.05), IE (*F*_(2,21)_=10.2; *P*<0.01), PPC (*F*_(2,21)_=7.0; *P*<0.01), PRh (*F*_(2,21)_=4.0; *P*<0.05) and OFC (*F*_(2,21)_=5.5; *P*<0.05), with no effect in the LE (*F*_(2,21)_=3.1; *P*>0.05). Duncan's post hoc tests revealed that Fos-positive counts were significantly greater following both Rev and IDrep shifting compared with cage mate controls in the mPFC, ME, IE, PPC, PRh and OFC (all *P*<0.05; Fig. 4c).

A targeted comparison of groups Rev and IDrep revealed that there was no difference in Fos-positive cell counts between these two behavioral groups in any brain region. There was an effect of brain region (*F*_(6,84)_=24.0; *P*<0.01), but no effect of behavioral group (*F*_(1,14)_<1; *P*>0.7), and no behavioral group × brain region interaction (*F*_(6,84)_<1; *P*>0.9). A focused comparision between mPFC and OFC, as conducted for Experiment 1, also failed to reveal any significant differences in Fos-positive cells as a function of behavioral group (Rev or IDrep) (task×brain region interaction: *F*_(1,14)_=0.05, *P*=0.83).

### Experiment 3

**Fig. 5:**
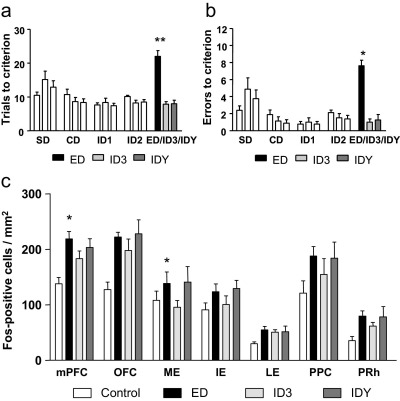
The number of trials taken (a) and the number of errors made (b) to reach a criterion of six consecutive correct trials on the attentional set-shifting paradigm. Animals from each group performed identical discriminations throughout SD, CD and intra-dimensional shifts 1 and 2 (ID1 and ID2). During the final discrimination animals performed either intra-dimensional shifts (ID3 and IDY) or an ED. Note that upon completion of the IDY, animals went on to complete the same number of trials as their yoked ED animal. Data are expressed as mean±SEM, *n*=8. * *P*<0.05 and ** *P*<0.01 significantly different from ID3 and IDY (Duncan's test). (c) The mean number of Fos-positive cell counts per mm^2^ tissue in the mPFC, OFC, ME, IE, LE, PPC and PRh from experimentally naïve cage-mate controls, animals which had performed an ED, an intra-dimensional shift (ID3) or animals which had performed an intra-dimensional shift in which the number of trials completed was yoked to the ED animals (IDY). Data are expressed as mean±SEM, *n*=8. * *P*<0.05 significantly different from ID3 (*t*-test), other statistics as detailed in results section.

As in Experiment 1, rats performing the ED shift discrimination during the final stage of Experiment 3 took significantly more trials and made significantly more errors than both groups of rats performing ID shift discriminations. One way ANOVAs revealed a significant effect of behavioral group on trials to criterion and errors during the final stage of the task (*F*_(2,21)_=44.9; *P*<0.01 and *F*_(2,21)_=42.8; *P*<0.01, respectively). Subsequent post hoc analysis revealed a significant difference between the number of trials to criterion and the number of errors made by rats performing an ED shift compared to those performing the ID shift discriminations (ID3 and IDY all *P*<0.05, Duncan's test; Fig. 5a, b). Note that on reaching criterion, rats performing the IDY discrimination then carried on to complete an equal number of trials to their yoked ED animal.

Overall, rats that performed more trials in the final discrimination test—groups ED and IDY—had higher Fos-positive cell counts relative to cage controls and rats that performed fewer trials in the final discrimination test (group ID3). Like Experiment 2, this finding is consistent with Fos being induced as a consequence of performing discrimination trials, rather than as a function of attentional demand. There was a significant effect of brain region (*F*_(6,168)_=75.6 *P*<0.01) and a significant effect of behavioral group (*F*_(3,28)_=5.1 *P*<0.01) on Fos-positive cell counts. However, there was no significant behavioral group×brain region interaction (*F*_(18,168)_<1.3; *P*>0.2). Further pairwise analysis of the main effect of behavioral group indicated that rats which had performed the ED and the IDY discriminations had significantly higher Fos-positive cell counts across all brain regions compared to cage-mate controls (*P*<0.05, Bonferroni). In contrast, those which had performed the ID shift but then stopped on reaching criterion (group ID3) did not differ from cage-mate controls (*P*>0.5) (Fig. 5c). Planned comparisons based on the outcome of Experiment 1 revealed significant differences in Fos-positive cells between groups ED and ID3 in mPFC (*t*_(14)_=1.83, *P*=0.044) and ME (*t*_(14)_=1.75, *P*=0.05), confirming that regional differences in Fos-LI that were seen in Experiment 1 were also observed in Experiment 3. Importantly, similar analyses comparing ED and IDY did not reveal any significant differences (all *P*>0.23).

### Effect of number of trials completed on Fos protein expression

**Fig. 6:**
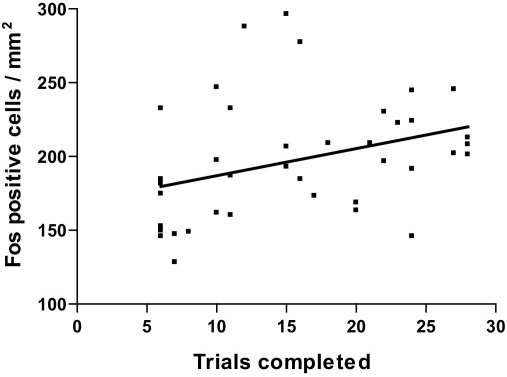
Positive correlation between Fos-positive cell counts in the mPFC and number of trials completed during the test discrimination in Experiments 1 and 3. *n*=40. *r*=0.348, *P*=0.027.

The pattern of the Fos-LI data suggested that the number of Fos-positive cell counts increased as the number of trials performed increased, irrespective of the type of trials performed. Therefore, the number of trials completed by rats in Experiment 1 and Experiment 3 were plotted against the Fos-positive cell counts from all brain regions. Data from Experiment 2 were not included because the experimental design was not comparable to Experiments 1 and 3. Specifically, stimuli in the Rev and IDrep discriminations were not novel, whereas the ED, ID3 and IDY discriminations involved new stimuli. A regression analysis demonstrated a significant positive correlation between the number of trials the rats completed during the test discrimination (irrespective of the type of trials performed) and their Fos-positive cell counts in the mPFC: (*r*=0.348, *P*=0.027; Fig. 6), but not in any other brain region (all *r*<0.13, *P*>0.42).

## Discussion

The aim of these experiments was to use IEG expression to examine the neuroanatomical systems activated by attentional and affective shifts in the rat brain. To achieve this aim, Fos-LI was used as a molecular marker of neural activation in specific cortical regions of the rat following completion of an attentional set-shifting paradigm which included ID shifts, ED shifts or reversals. Fos-LI increased in the mPFC following ED shifting, relative to rats that learned a new ID shift to criterion, as well as in medial and intermediate divisions of the entorhinal cortex. This is consistent with the finding that lesions of mPFC and entorhinal cortex impair ED shifting and attentional set formation, respectively (Birrell and Brown, 2000; Oswald et al., 2001). However, a subsequent experiment found that this increase of Fos-LI in the ED shift condition could be accounted for by the increased number of trials that were required to reach criterion in this condition; rats that worked a yoked number of additional ID shift trials after reaching criterion showed equivalent levels of Fos-LI in the mPFC to rats in the ED shift condition. Indeed, the levels of Fos-LI in various cortical regions following performance of ED shifts, ID shifts and reversal discriminations were more closely related to the number of trials the rats performed than any differences in the cognitive demands of the discriminations.

In Experiment 1, rats which had completed an ED shift had significantly greater Fos-positive cell counts across all brain regions than those which had completed an ID shift. This effect was strongest in the mPFC and the medial and intermediate subregions of the entorhinal cortex, and was significantly stronger in mPFC than in OFC. This is consistent with the effects of lesions of these two areas on performance of ED shifts (Birrell and Brown, 2000; McAlonan and Brown, 2003). Importantly, the design of the task was such that the two behavioral groups (ED and ID) were exactly matched for their previous history and for the final discrimination, in terms of motor activity and exposure to novel sensory stimuli, with the exception of a difference in the requirement to shift attentional set in the ED group. There was no indication that GABAergic cortical interneurons were specifically engaged by any aspect of the behavioral tasks. This suggests that although dysfunction of the GABA-ergic system may contribute to deficits in attentional set-shifting (Pratt et al., 2008), they are not activated to the same extent as other cortical neurons in this type of behavioral task.

However, there was a limitation in the design of Experiment 1, in that rats which performed the ED shift took more trials and made more errors before reaching criterion than those performing the ID shift, resulting in a large discrepancy between the overall task activity of the two groups before determination of Fos-LI. Of course, this is unavoidable given the difference in difficulty between ED and ID shifts, a feature of the discrimination paradigm that validates it as a test of attentional shifting. Therefore, although it was possible that the increase in Fos-LI in rats that had performed ED shifts reflected cognitive demand, it could also have been related to task activity. As noted in the introduction, this is a problem with other experiments that have compared gene expression after different phases of the task (Glickstein et al., 2005; DeSteno and Schmauss, 2008). Other investigators have used control conditions in which no discrimination learning was involved (Tait et al., 2009), which hardly seems appropriate to us either. Experiments 2 and 3 were designed to address the possibility that changes in gene expression were related to task activity instead of cognitive demand. In these experiments, two further control groups were introduced: a group of behaviorally naive cage-mate control rats to provide an indication of basal Fos expression with minimal cognitive activity, and a group of rats which performed the final ID shift but then went on to complete a comparable number of trials as the rats performing the test discrimination (ED or Rev), thereby being yoked in terms of overall task activity. It was not possible to also yoke the animals in terms of the number of errors made, without introducing a confound in terms of omission of expected rewards.

In Experiment 2, rats which had performed the Rev discrimination had significantly higher Fos-positive cell counts in all brain regions examined except the lateral entorhinal cortex relative to untested cage-mate controls. However, there were no significant differences between Fos-LI in rats which had performed the Rev discrimination and rats in the control condition that had repeated the ID shift and completed the same number of trials as their yoked Rev partner. This behavioral control condition differed from that used in Experiment 1 because the reversal, unlike the ED shift, uses stimuli that are already familiar to the rat. Thus, even though the reversal was more difficult than learning the discrimination initially, we did not observe any specific Fos activation associated with the discrimination reversal. This unexpected result suggested that the increase in Fos-LI might be more closely related to the number of trials that the rats had performed rather than the actual cognitive demands of the trials.

Experiment 3 tested whether the increased number of trials performed by rats during the ED shift, rather than the nature of the task, explained the increase in Fos-LI seen in Experiment 1. This experiment used an additional control group, group IDY, who received an ID shift in the final test phase and then completed an equal number of trials as group ED, by yoking individual rats in the two groups. Thus, both groups ED and IDY encountered a novel discrimination problem in the final test phase, but group IDY did not have to engage an attentional shift to solve the discrimination efficiently. Equivalent levels of Fos-LI were observed in all areas measured in groups ED and IDY. This is consistent with the finding from Experiment 2 and suggests that Fos-LI is more related to behavioral activity than to specific cognitive demands. Consistent with this conclusion, there was a significant correlation between the number of trials performed by each rat during the final discrimination (ED, IDY, or ID3) in Experiments 1 and 3, and Fos-positive cell counts in the mPFC. These data indicate that Fos-immunoreactivity in the mPFC is more closely related to the number of trials rats performed than the differing cognitive demands of the trials across discriminations. The time course of Fos expression relative to stimulation is sufficiently gradual (e.g. Hughes and Dragunow, 1995) that it is difficult to account for our results in terms of, for example, differential decay of Fos between our behavioral groups.

The importance of matching control conditions for all sensory and motor aspects of a task has recently been emphasized in a study examining the effect of spatial learning in the Morris water-maze on IEG expression (Shires and Aggleton, 2008). These authors found no difference in hippocampal Fos-LI between rats that performed a hippocampal-dependent spatial learning task, and those that performed a hippocampal-independent procedural control task. This result suggests that previous studies which have observed increases in IEG expression in the hippocampus in response to spatial learning in a water-maze may not have been using appropriate controls to determine changes in gene expression related specifically to learning (Guzowski et al., 2001; Teather et al., 2005). Consistent with this view, rats trained in brightness discrimination in a Y-maze showed very similar patterns of c-fos mRNA expression in the hippocampus and cerebral cortex to rats that had experienced unpaired presentations of the same sensory stimuli and reinforcers (Grimm and Tischmeyer, 1997). These considerations also suggest that differences in Egr-2 expression between mice trained in set-shifting and comparison groups that foraged for food, without discrimination training, may not be specific to attentional demand (DeSteno and Schmauss, 2008).

Many cognitive components are involved in ED set-shifting, such as recognition of both correct and incorrect feedback, selection of a new dimension, response inhibition and retention of attentional set (Konishi et al., 1999). However, all but the selection of a new dimension are also required to some degree for ID shifting, making it very difficult to identify the neural substrates of increased cognitive demands imposed by ED set-shifting. The increase in Fos expression observed in the present series of experiments may reflect the volume of sensory input and processing involved in the attentional set-shifting paradigm, rather than the outputs of specific brain regions and thus their necessity for the execution of ED set-shifting. The most striking observation in this regard was a significant correlation between the number of trials performed and Fos-LI in mPFC, which was not found in any other brain area examined. This would be congruent with a role for the PFC, specifically dorsolateral PFC, in monitoring of sensory stimuli that are currently behaviorally-relevant (Petrides, 1998), a role which may be performed by the mPFC in rats. In line with this theory, a recent study examining the trajectories of simultaneously recorded mPFC neurons during a set-shifting task found that the mPFC continues to maintain an online representation of a rule even once the rule has been learnt and the information is no longer relevant for responding (Durstewitz et al., 2010). This may reflect continuous monitoring by the mPFC to detect potential rule violations and could account for the increase in Fos expression in the current study. Fos induction in this area may also reflect the activation of memory processes for particular task strategies or attentional sets (Rich and Shapiro, 2007), that are continuously updated during task performance.

Fos expression is considered to be related to neuronal activation rather than to synaptic plasticity per se (e.g. Alberini, 2009). This was our rationale for choosing Fos as a marker in this study—we were interested in mapping neuronal activation in response to demands on different aspects of executive function (affective and attentional shifting) that have been associated with different regions of PFC. It is possible that differences in gene expression would be revealed with other IEGs, such as *arc* (Bramham et al., 2008) that are more closely associated with synaptic plasticity. We did not attempt to determine other genes or gene products in this experiment because their time course of expression is not consistent with determining Fos, but this would be an interesting topic for future research. Importantly, our results suggest that behavioral testing in advance of measuring gene expression should control for the number of trials animals are exposed to, in addition to the cognitive demands.

## Conclusion

In contrast to the conclusions drawn from previous lesion and neuroimaging studies, the current data suggests that the mPFC and other structures required for efficient attentional shifting, like the entorhinal cortex, do not remain quiescent until a shift in attentional set is required. Neural activity in these structures, measured by an increase in Fos-LI, increases during discrimination learning and performance even when shifts in attention are not required. Thus, we speculate that aberrant neural activity within the mPFC, as a consequence of disrupted input from other cortical areas or abnormal neuromodulation of this area, may be more detrimental to behavior than the selective effects of mPFC ablation or inactivation on attentional shifting might suggest.

## Figures and Tables

**Table 1:** Example of a sequence of discriminations in the attentional set-shifting paradigm

Discrimination	Odour pair	Medium pair
Simple discrimination	Cinnamon/cumin	Bedding
Compound discrimination	Cinnamon/cumin	Cat litter/ground cat litter
Intradimensional shift 1	Nutmeg/coriander	Pebbles/gravel
Intradimensional shift 2	Mint/paprika	Sawdust/shavings
Extradimensional shift	Cloves/thyme	Wax chips/ground wax

Rewarded exemplars for a rat undergoing an odour to medium extradimensional shift are underlined.

**Table 2:** Colocalisation of GAD67 and Fos-like immunoreactivity in the medial prefrontal cortex (mPFC), orbitofrontal cortex (OFC), medial entorhinal cortex (ME), intermediate entorhinal cortex (IE), lateral entorhinal cortex (LE), perirhinal cortex (PRh) and posterior parietal cortex (PPC) of rats which had performed extra-dimensional (ED) or intra-dimensional (ID3) shifts in Experiment 1

Brain region	ED	ID3
mPFC	11.31±1.34	14.44±2.68
OC	9.12±1.77	6.08±1.52
ME	2.85±1.09	2.28±0.81
IE	0.38±0.25	0.38±0.25
LE	0±0	0±0
PrH	11.40±1.72	7.22±1.37
PPC	17.10±2.91⁎	9.12±1.91

Data are expressed as mean number of double-labelled cells per mm^2^ tissue ± SEM, *n*=8 per group.

⁎ *P*<0.05 significantly different from ID3.
